# Supplementary material for: The genome sequence of the chaffinch, Fringilla coelebs Linnaeus, 1758
Source: Wellcome Open Res. 2024 Apr 18;9:207. [Version 1] doi: 10.12688/wellcomeopenres.21279.1 (PMC11303953; doi:10.12688/wellcomeopenres.21279.1)
Supplement: Peer review history

# Reviewer report: Reviewer response for version 1

**DOI:** 10.21956/wellcomeopenres.23535.r88446

**Published:** 2024-08-09

**Authors:** Zhengchang Su, Siwen Wu

**Affiliations:** 1 Department of Bioinformatics and Genomics, University of North Carolina at Charlotte, Charlotte, USA; 2 The University of North Carolina at Charlotte, Charlotte, North Carolina, USA

This study assembled the genome of a *Fringilla coelebs* male individual using PacBio HiFi long reads for contig assembling and Hi-C reads for scaffolding. The final assembly is at chromosome-level with relatively high quality. However, I have a few concerns for the authors to address before the paper is accepted for Indexing.

 1) Why did the authors use a male individual to assemble the genome? A female would allow the assembling for both W and Z sex chromosomes.

 2) Parameters used in each tool should be included in the methods section or indicating default settings were used.

 3) More aspects of assessment of the assembly should be included, such as the reliable block N50 and genome heterozygosity.

 4) Percentage of the assembly mapped to chromosomes (90.11%) is lower than  the benchmark indicated in Table 1 (95%). What caused the relatively low number?

 5) How is the number of chromosomes in the bird determined?

Are sufficient details of methods and materials provided to allow replication by others?

Yes

Is the rationale for creating the dataset(s) clearly described?

Yes

Are the datasets clearly presented in a useable and accessible format?

Yes

Are the protocols appropriate and is the work technically sound?

Yes

Reviewer Expertise:

Genomics, computational biology, bioinformatics

We confirm that we have read this submission and believe that we have an appropriate level of expertise to confirm that it is of an acceptable scientific standard, however we have significant reservations, as outlined above.

# Reviewer report: Reviewer response for version 1

**DOI:** 10.21956/wellcomeopenres.23535.r88444

**Published:** 2024-08-06

**Authors:** Merly Escalona

**Affiliations:** 1 Department of Biomolecular Engineering, University of California Santa Cruz, Santa Cruz, California, USA

In this study, the authors present a high-quality genome assembly of the chaffinch. The genome was assembled using PacBio HiFi long reads and Hi-C data, followed by manual curation. The methods used for data generation, processing, assembly, and quality checks are well-documented and described adequately. Assembly metrics satisfy most of the benchmarks for the VGP-2020 baseline (Rhie et al. 2021). The metadata provided is comprehensive.

 The main comment would be to state the rationale for assembling the genome more clearly in the introduction. While the authors provide valuable ecological context for the bird, the text could be strengthened by briefly summarizing any existing genetic work on the species (or lack thereof), apart from being a target species for the Darwin Tree of Life project. 

 Minor comments: 

1. In the background, the third sentence, “ *The genus name Fringilla, Latin for ‘small bird’, can be traced back to ancient authors,*” could either be omitted, as it does not provide useful information, or alternatively, rewritten to provide more specific details about the etymology. ‘Ancient authors’ is ambiguous. 
    
2. In sentence 4 of the background, replace ‘ *upperpart colouration’* with ‘ *colouration of the upper part of the head.*'
3. In the third paragraph of the background, edit: “ *Chaffinches breed in wooded areas, parks, and gardens from mid-April to mid-July. Only females incubate the eggs, but both parents feed the nestlings.*”
4. In the section Genome sequence report, please change the first sentence to: “ *We sequenced the genome of a male..*”
5. In the section Genome sequence report, replace ‘ *number*’ with ‘ *reducing the proportion of scaffolds’* in the sentence: “ *Manual assembly curation corrected 50 missing joins or mis-joins, reducing the scaffold number by 7.07%.*”
6. Please be more specific with the genome assembly metrics presented in Table 1. 
  1. Do the values for the “Assembly metrics” section in Table 1 correspond to the primary haplotype of the assembly, or do they represent values for the diploid assembly?
  2. Are the metrics for the “Genome assembly” section in Table 1 for the primary haplotype?   

Are sufficient details of methods and materials provided to allow replication by others?

Yes

Is the rationale for creating the dataset(s) clearly described?

Partly

Are the datasets clearly presented in a useable and accessible format?

Yes

Are the protocols appropriate and is the work technically sound?

Yes

Reviewer Expertise:

genome assembly, bioinformatics

I confirm that I have read this submission and believe that I have an appropriate level of expertise to confirm that it is of an acceptable scientific standard.

# Reviewer report: Reviewer response for version 1

**DOI:** 10.21956/wellcomeopenres.23535.r88442

**Published:** 2024-08-06

**Authors:** Leif Andersson

**Affiliations:** 1 Department of Veterinary Integrative Biosciences, Texas A&M University, College Station, Texas, USA

This report provides a high-quality assembly of the chaffinch which is an abundant passerine in Europe and Western Asia. The methods and resulting data are well documented. This is an important resource for genomic studies of this species.

 I assume that chromosomes have been numbered according to the length of the chromosomal pseudomolecules but that should be stated. Also, it would be of interest to provide information on the correspondence between these chromosome numbers in chaffinch and chromosome numbers in the zebra finch, a well-studied genome.

 I also have one minor comment and that it would be nice to include the correct citation to Linnaeus first description of the species.

Are sufficient details of methods and materials provided to allow replication by others?

Yes

Is the rationale for creating the dataset(s) clearly described?

Yes

Are the datasets clearly presented in a useable and accessible format?

Yes

Are the protocols appropriate and is the work technically sound?

Yes

Reviewer Expertise:

Evolutionary genomics

I confirm that I have read this submission and believe that I have an appropriate level of expertise to confirm that it is of an acceptable scientific standard.

# Reviewer report: Reviewer response for version 1

**DOI:** 10.21956/wellcomeopenres.23535.r88438

**Published:** 2024-08-06

**Authors:** Jérôme Fuchs

**Affiliations:** 1 Institut de Systématique Evolution Biodiversité, Muséum national d'Histoire naturelle CNRS SU EPHE UA, Paris, France

The following manuscript present a chromosome level assembly of the Chaffinch, a common woodland bird species in the Western Palaearctic.

 The amount of data and quality of the assembly is, without any doubt, in line with current standard. I have a few comments that the authors may consider.

- "Most (90.11%) of the assembly sequence was assigned to 40 chromosomal-level scaffolds, representing 39 autosomes and the Z sex chromosome."
- 2n= 80 corresponds to the number of diploids chromosomes for the Chaffinch; maybe worth mentioning in the manuscript

it is my understanding that the remaining contigs/scaffolds were unassigned; since the tissue used for sequencing was heart, is there any possibly that unassigned contigs are attributable to blood parasites ? or is mostly repeat sequences ?

 As part of a genome description publication, it may be useful to further comparative analyses have the proportion of:

- the percentage of repeat sequences
- number of snps in the genome and per chromosome
- number of protein coding genes

It would also be worth comparing this genome assembly with the one assembled by Recuerda et al. (2021; doi.org/10.1093/gbe/evab034) for the same species (synteny plot, number of CDS ?).

Are sufficient details of methods and materials provided to allow replication by others?

Yes

Is the rationale for creating the dataset(s) clearly described?

Yes

Are the datasets clearly presented in a useable and accessible format?

Yes

Are the protocols appropriate and is the work technically sound?

Yes

Reviewer Expertise:

Avian systematics and evolution

I confirm that I have read this submission and believe that I have an appropriate level of expertise to confirm that it is of an acceptable scientific standard, however I have significant reservations, as outlined above.

# Reviewer report: Reviewer response for version 1

**DOI:** 10.21956/wellcomeopenres.23535.r88440

**Published:** 2024-07-04

**Authors:** Luohao Xu

**Affiliations:** 1 MOE Key Laboratory of Freshwater Fish Reproduction and Development, School of Life Sciences, Southwest University, Chongqing, Chongqing, China

The chaffinch is an ecologically and evolutionarily important songbird. I am glad its genome is now assembled at the chromosome level with high quality. I only have a few minor comments.

 The authors determined 40 chromosome models, but it is unclear how it was done. Was it just selecting the top 40 largest scaffolds, or using zebra finch/chicken chromosomes as a reference?

 The hifiasm version (0.16.1-r375) is a bit outdated. The current version is 0.19.9-r616.

Are sufficient details of methods and materials provided to allow replication by others?

Yes

Is the rationale for creating the dataset(s) clearly described?

Yes

Are the datasets clearly presented in a useable and accessible format?

Yes

Are the protocols appropriate and is the work technically sound?

Yes

Reviewer Expertise:

Evolutionary genomics

I confirm that I have read this submission and believe that I have an appropriate level of expertise to confirm that it is of an acceptable scientific standard.
